# Predictive factors for persistent thrombocytopenia after peptide receptor radioligand therapy in enteropancreatic neuroendocrine tumors

**DOI:** 10.3389/fendo.2025.1568243

**Published:** 2025-05-15

**Authors:** Romain Ferrara, Christophe Zemmour, Thibaut Reichert, Daniel Ouk, Patricia Niccoli, Jemima Maniry-Quellier, Isabelle Brenot-Rossi, Nathalie Charrier, Sandrine Oziel-Taieb

**Affiliations:** ^1^ Department of Nuclear Medicine, Institut Paoli-Calmettes, ENETS Center of Excellence, IPC NET Center, Marseille, France; ^2^ Department of Clinical Research and Investigation, Biostatistics and Methodology Unit, Institut Paoli-Calmettes, Marseille, France; ^3^ Department of Medical Oncology, Institut Paoli-Calmettes, ENETS Center of Excellence, IPC NET Center, Marseille, France

**Keywords:** [177Lu]Lu-DOTATATE, neuroendocrine tumors, thrombocytopenia, bone metastases, spleen

## Abstract

**Introduction:**

Peptide receptor radionuclide therapy (PRRT) is an effective and well-tolerated treatment for advanced neuroendocrine tumors (NETs). However, persistent thrombocytopenia (PT) has been reported and may compromise further therapies and outcomes. This study aimed to identify predictive factors for PT defined as a platelet count <100 x 10^9^/L, 2 months after the end of PRRT.

**Methods:**

We performed a single-center retrospective analysis of clinical, biological, and imaging parameters of metastatic NET patients undergoing [177Lu]Lu-DOTATATE therapy. Bone metastatic volume was quantitatively measured and converted into an Osteo-Medullary Invasion Score (OMIS). The initial decline of platelet count (IDPC) was defined as the relative change (%) in platelet count between the baseline and the nadir value before the second cycle.

**Results:**

In total, 47 patients (25 women, 22 men, median age 68 years) were included. Fifteen patients (31.9%) had bone metastases, and five (10.6%) had an OMIS ≥ 30%. Six patients (15.4%) presented with a spleen length ≥ 100 mm. Median follow-up was 50.1 months. Median IDPC was 26%. Eight patients (17%) presented with PT. PT was associated with an OMIS ≥ 30% (p < 0.001; odds ratio not estimable), a spleen length ≥ 100 mm (p = 0.04; odds ratio = 7), and an IDPC ≥ 30% (p= 0.014: odds ratio = 15.8), and was unrelated to age, gender, previous cancer, previous therapies, and cumulative activity.

**Conclusion:**

We found that 17% of PT incidence correlated with relatively high bone metastatic burden and spleen length. Physicians should be vigilant in the event of a significant drop in platelet count after the first cycle of PRRT.

## Introduction

Neuroendocrine tumors (NETs) are relatively rare neoplasms originating from endocrine cells, primarily in the gastroenteropancreatic tract and the pulmonary system. Due to their slow natural course, NETs are identified as locally advanced or with distant metastasis in 40%–50% of patients. Patients with metastatic NETs often have a prolonged survival, justifying precautions in terms of persistent treatment-related toxicity (1, 2). Peptide receptor radionuclide therapy (PRRT) has emerged as a crucial strategy for treating advanced NETs (3) through systemic administration of radiolabeled somatostatin analogs to selectively target cancer cells expressing somatostatin receptors (SSTRs). The compound is internalized by endocytosis and retained in the lysosomes of cells, allowing the delivery of cytotoxic radiation directly to target cells and therefore leading to the breakdown of intracellular DNA chains and cell death. The standard schedule for PRRT consists of four infusions of 7.4 GBq (200 mCi) [177Lu]Lu-DOTATATE every 8 weeks, extended up to 16 weeks in case of toxicity (4). PRRT can induce hematological toxicity (HT), typically mild and reversible, that results from irradiation of hematopoietic tissue. Up to 10% of patients may develop grade 3/4 HT, and thrombocytopenia is the most common cause of dose reduction or treatment interruption (5, 6). Additionally, there is a related long-term risk of 2% for the development of myelodysplastic syndrome and 1% for acute myeloid leukemia after PRRT (3, 7). These malignancies are very often preceded by persistent thrombocytopenia (PT) (8, 9). In the event of early use of PRRT in the NET therapeutic arsenal, prolonged PT after the end of PRRT may compromise the patients’ access to other therapeutic alternatives and thus worsen the prognosis. Predictive factors for persistent cytopenia post-PRRT are not well-defined, and hematological toxicity data are mainly retrospective with heterogeneous populations in terms of metastatic spread, particularly bone involvement. However, patients with widespread bone metastases are at greater risk of acute HT and persistent cytopenia (10–12). Anecdotally, a splenectomy appears to have a beneficial effect, possibly due to the presence of SSTRs on lymphocytes, nonetheless, published data is extremely sparse (6, 13). In a previous pilot study, which included a small population of 20 patients, we identified three potential predictive factors for PT using biological and imaging derived parameters: the percentage of osteo-medullary invasion, the length and volume of the spleen, and the percentage of reduction in platelet count between the first two cycles of [177Lu]Lu-DOTATATE (14). The aim of this retrospective study is to ascertain potential predictive factors for PT in a larger population of 47 metastatic NET patients, including the initial 20.

## Methods

### Patients

Between June 2016 and August 2022, 47 patients with metastatic NETs whose indication for PRRT was validated by the NET multidisciplinary tumor board (RENATEN) were treated with [177Lu]Lu-DOTATATE at the ENETS Center of Excellence of Paoli-Calmettes Institute. Patients were treated with [177Lu]Lu-DOTATATE if the tumor uptake was at least as high as the uptake in the normal parenchyma of the liver on 111In-DTPA-octreotide scintigraphy (OCTREOSCANR) or 99m TC-EDDA/HYNIC-TOC (TEKTROTYDR), or 68Ga-DOTA-TOC prior to the therapy. Eligibility criteria were: platelets ≥ 100 x 10^9^/L, hemoglobin ≥ 8 G/dL, leukocytes ≥ 2 x 10^9^/L, absolute neutrophil count ≥ 1 x 10^9^/L, creatinine clearance ≥ 40 mL/min, bilirubin < 3 N, albumin ≥30 g/L, and prothrombin rate ≥70%. The study was conducted after approval by our local institutional review board (PREDIRIV-IPC 2022-003). For this retrospective analysis, the institutional review board approved a waiver for informed consent.

### Treatment

A maximum of 7.4 GBq (200 mCi) [177Lu]Lu-DOTATATE per cycle was administered with an interval of at least 8 weeks, aiming for a maximum of four cycles and a cumulative activity of 29.6 GBq (800 mCi). A concurrent infusion of Aminoven 5% or 2.5% arginine and 2.5% lysine saline (since 2018) was administered to limit renal toxicity. Hematopoietic growth factors were not used. Radio-sensitizing chemotherapy was not associated with [177Lu]Lu-DOTATATE.

### Biological toxicity assessment

For all the included patients, routine laboratory tests including hematological, liver, and renal function tests were obtained before the start of PRRT; 1 day before each treatment cycle; 2, 4, 6, and 8 weeks after each cycle; and during the follow-up visits every 4 to 6 months after completion of PRRT. The platelet nadir values between the PRRT cycles were retrieved for the analyses in this study. The initial decline of platelet count (IDPC) is defined as the relative change (in percentage) in platelet count between the baseline and the nadir value before the second cycle. The Common Terminology Criteria for Adverse Events (CTCAE) version 5.0 was applied to score platelet toxicity. We defined a PT as a platelet count <100 x 10^9^/L 2 months after the end of PRRT. This cut-off was chosen as we considered it particularly relevant given our standard caution for further antitumoral therapy administration.

### Post-PRRT [177Lu]Lu-DOTATATE scintigraphy

Post-PRRT [177Lu]Lu-DOTATATE scintigraphy was performed after each administration to check tumor targeting, using a Discovery NM/CT 670 hybrid gamma camera (General Electric Medical^®^) equipped with a medium-energy collimator, with the energy window centered on 208 keV. Whole-body planar scan acquisitions were carried out at a scan speed of 10 cm/min using a 1024 × 256 matrix. Single-photon emission computed tomography (SPECT) acquisitions were performed systematically, exploring the abdominal and pelvic regions at least. Each 180° rotation was performed with a 6° angle increment, providing 60 projections acquired with both heads (128 x 128 matrix). Coupling to CT images was obtained by acquisitions made immediately prior to SPECT acquisition, maintaining the same field limits. SPECT images were reconstructed using the OSEM reconstruction algorithm supplied by the manufacturer (Xeleris 3.0), with two iterations and ten subsets. Attenuation correction was based on the attenuation map obtained from the CT scan. Up until 2020, for the first 25 patients, post-PRRT [177Lu]Lu-DOTATATE scintigraphy was performed on Day+1 and since 2020, at the 4th hour.

### Quantitative assessment of the osteo-medullary invasion: the Osteo-Medullary Invasion Score

**Figure 1:**
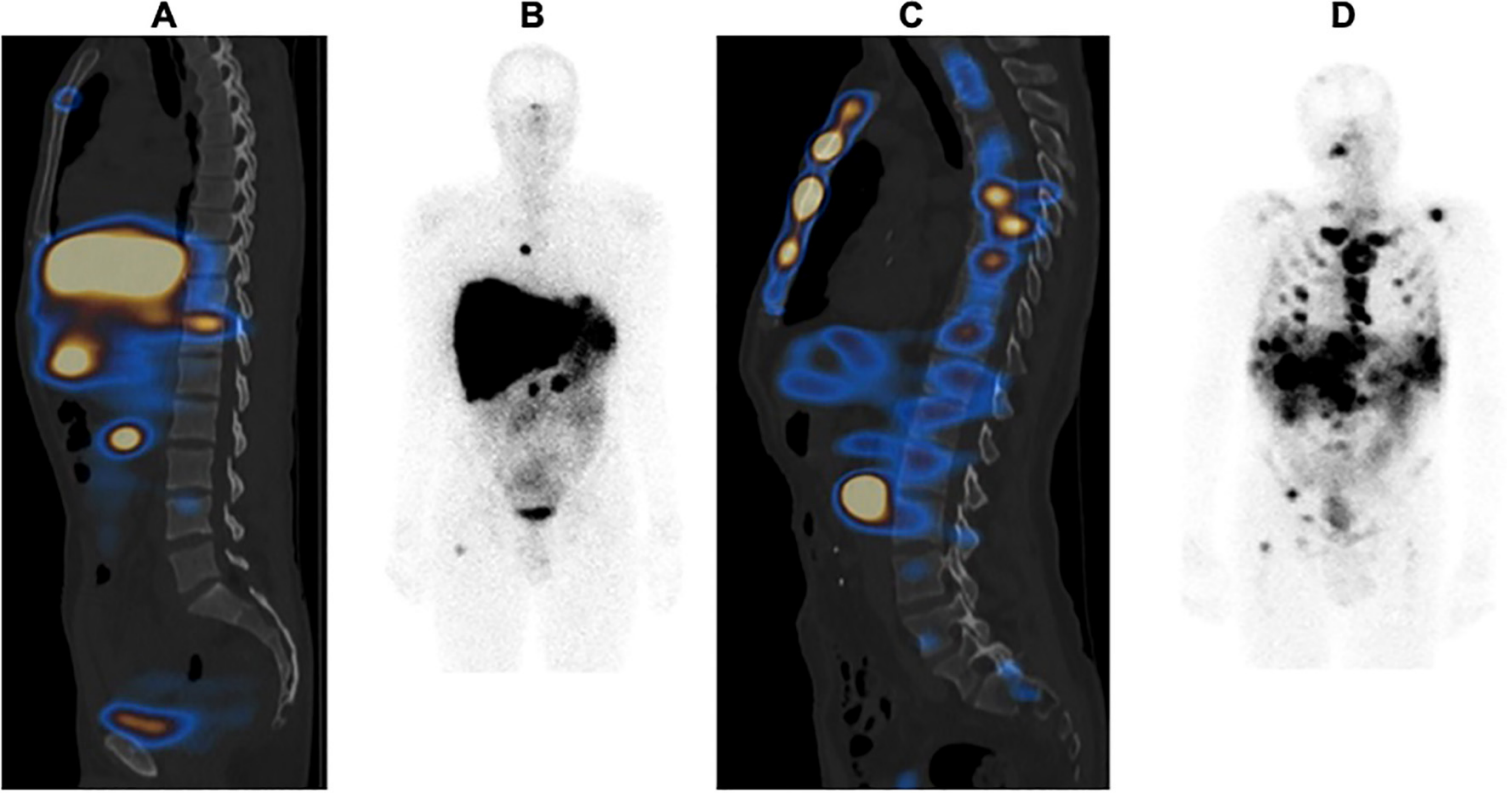
Example of an Osteo-Medullary Invasion Score (OMIS) in two different patients: **(A, B)** patient with 13% OMIS; **(C, D)** patient with 80% OMIS.

Based on the work of Cristy (15), which described the distribution of active bone marrow (in percentages) in each bone of the skeleton of a 40-year-old man, an approximate weighting factor was assigned to each bone type. Observable bone lesions were counted using the first post-PRRT [177Lu]Lu-DOTATATE scintigraphy. As soon as a lesion was detected, the entire bone was considered to be affected, and the corresponding score was assigned. For example, in the case of bone metastasis of two lumbar vertebrae, the OMIS was estimated at 5%, as all five lumbar vertebrae represent 12.3% of the active bone marrow and each lumbar vertebra represents approximately 2.5% of the active bone marrow. The OMIS was the total sum of the scores (**Figure 1**).

### Spleen length assessment

**Figure 2:**
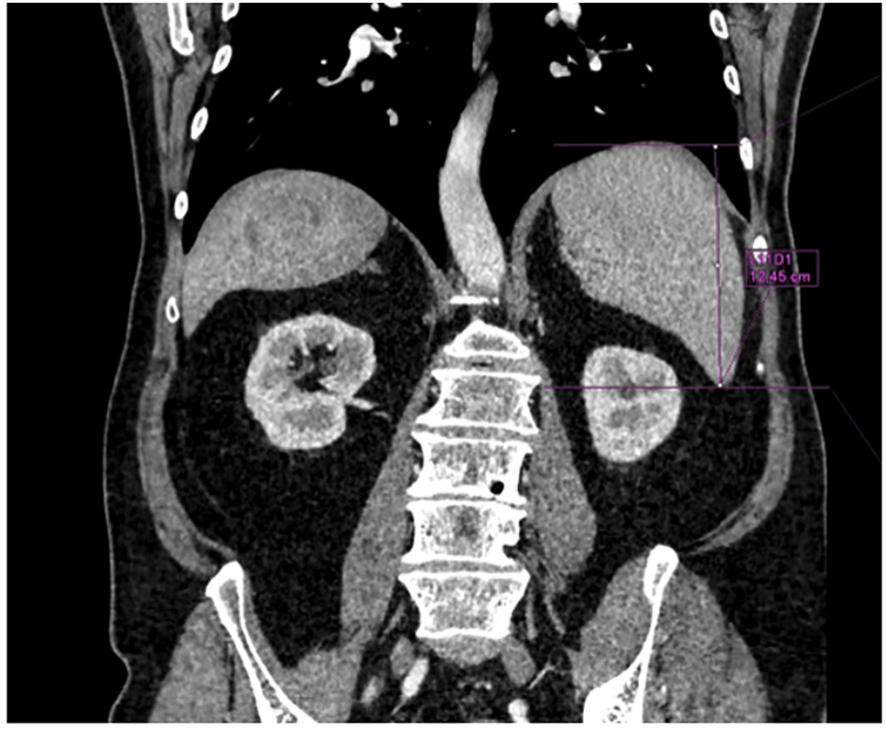
Example of spleen length measurement with Syngo®.via (Siemens Healtineers).

The most recent morphological imaging data (abdominal MRI or CT), within 2 months before PRRT, was used to measure the splenic length as the greatest vertical spleen dimension on coronal view; Syngo^®^. Via (Siemens Healthineers) software was used (**Figure 2**). We considered 100 mm to be the upper limit of normality for the spleen length (16).

### Statistical analyses

Statistical analyses were performed at the significance level α=0.05 with the SAS^®^ 9.4 software. Patients and treatment characteristics were described using counts and frequencies for categorical endpoints, and medians (ranges) for quantitative endpoints. The prognostic impact on PT occurrence of baseline potential predictive factors was assessed in univariate analyses, e.g., Fisher’s exact tests or univariate logistic models. Wilcoxon tests were used to compare quantitative patients’ characteristics depending on PT occurrence. Odds ratios (ORs) of factors significantly associated with PT occurrence, or close to the significance, were estimated with their bilateral Wald confidence intervals. The area under the curve (AUC) and receiver operating characteristic (ROC) were estimated with their Wald bilateral confidence intervals for quantitative endpoints. Overall survival was defined as the time from treatment initiation to death. Progression-free survival was defined as the time from treatment initiation to progression or death. Patients without considered events were right-censored at their last follow-up. Median survivals were estimated using the Kaplan–Meier method with their bilateral confidence interval. Follow-up was estimated using the reverse Kaplan–Meier method.

## Results

### Patient baseline characteristics

A total of 53 patients with metastatic enteropancreatic NETs were treated with PRRT between June 2016 and August 2022. We selected the patients who had received at least two PRRT cycles and for whom laboratory data were available for both the baseline value and at least 2 months after the final PRRT cycle. Six patients were excluded (only one PRRT cycle for five patients). Thus, 47 patients met the inclusion criteria for this study. Their median age was 68 years (range 25–84 years). The primary tumor was predominantly a small intestine NET (n=42, 89.4%). Furthermore, 17 patients had a grade 1 NET (Ki67<3%) and 30 patients a grade 2 (Ki67: 3%–20%). The median Ki67 was 5% (range 0.5–20). Seven patients had a previous cancer (14.9%): two patients with prostate cancer, three patients with breast cancer, and one with Hodgkin lymphoma. Moreover, 12 patients (25.5%) had been previously treated with chemotherapy (alkylating agents: 4) and 7 patients (14.9%) with external-beam radiation therapy (EBRT). Eight patients (17%) had received targeted therapies before PRRT (everolimus and sunitinib). Bone metastases were present in 15 patients (31.9%). The median baseline glomerular function rate (GFR) was 79 ml/mn (range 40–107). The median baseline platelet count was 222 x 10^9^/L (range 108–486). In total, 38 patients received all the planned four cycles of [177Lu]Lu-DOTATATE (80.8%), 6 patients received three cycles (12.8%), and 3 patients received two cycles (6.4%). The median cumulative administered activity of [177Lu]Lu-DOTATATE was 29.1 GBq (range 12.6-30.1). Median follow-up was 50.1 months [95% confidence interval (33.5–63.2)], median progression-free survival was 27.4 months [95% confidence interval (20.9–33.6)], and median overall survival was 43.3 months [95% confidence interval (32.7–not estimable)]. Among the population with bone metastasis, the median OMIS was 26.5% and the mean OMIS was 29.8%. Six patients (15.4%) presented with splenomegaly, defined as a spleen length larger than 100 mm, and one patient had a previous splenectomy. The full patient characteristics are summarized in **Table 1**.

**Table 1:** Baseline characteristics of the 47 metastatic NET patients.

Characteristic (n)	Number (%)
Overall	47 (100)
Gender : Female/Male	25 / 22 (53.2/46.8)
Median age (y)	68 (range 25-84)
Neuroendocrine tumors location (n)	
Small intestine	42 (89.4)
Pancreas	4 (8.5)
Rectum	1 (2.1)
WHO Grade (n)	
Grade 1	17 (36.2)
Grade 2	30 (63.8)
Median Ki67 (%)	5 (range 0.5-20)
Bone metastases	15 (31.9)
Pelvic bone metastases	12 (25.5)
Osteo-Medullary Invasion Score OMIS 30%	5 (10.6)
Prior treatments	
Chemotherapy	12 (25.5)
External-beam radiation therapy	7 (14.9)
Targeted therapy	8 (17)
Median platelet count before PRRT X 10^9^/L	222 (range 108-486)
Median baseline GFR (ml/min)	79 (range 40-107)
Spleen length > 100 mm	6 (15.4)
Median [^177^Lu]Lu-DOTATATE Cumulative activity (GBq)	29.1 (range 12.6-30.1)

### Subacute and persistent thrombocytopenia

Eight of the 47 patients had PT after [177Lu]Lu-DOTATATE therapy (17%). Two of them received prior alkylating agents and three of them were previously treated with EBRT. None of them had baseline renal dysfunction. Five of them had bone metastases with an OMIS ≥ 30% (range 0–90). Three of them had a spleen length ≥ 100 mm and also a baseline platelet count < 150 x 10^9^/L. [177Lu]Lu-DOTATATE was prematurely discontinued (less than four cycles) in five patients due to PT. Cumulative [177Lu]Lu-DOTATATE activity did not exceed 29.5 GBq in this group. Five of them (62.5%) had a platelet count lower than 100 x 10^9^/L 1 year after the end of PRRT. The characteristics of the eight patients who developed PT are detailed in **Table 2**. Patient no. 1 developed a complex hematological malignancy 40 months after the end of PRRT that was associated with a myelodysplastic syndrome with an IgG Lambda multiple myeloma. Metastatic neuroendocrine cells were also present in the bone marrow sample. He died 53 months after the end of PRRT from disease progression. Patient no. 43, a 69-year-old man, developed severe pancytopenia without dysplasia but with bone marrow infiltration by neuroendocrine cells and died 11 months after the last cycle of PRRT with diffuse progressive disease. Patient no. 2, a 59-year-old man, did not recover from PT and presented with bone marrow hypoplasia without dysplasia. He died 12 months after the end of PRRT due to progressive disease and cardiac insufficiency. Patient no. 28, a 75-year-old man who first recovered from PT, was rechallenged with a single cycle of PRRT because of progressive disease and then developed definitive severe thrombocytopenia. All eight patients with PT died from progressive disease. There was no thrombocytopenia-related death. No patient developed acute leukemia.

**Table 2:** Characteristics of the 8 patients with persistent thrombocytopenia after PRRT.

Patient n°	1	2	13	14	20	28	42	43
Gender	M	M	M	M	F	M	F	F
Age (y)	75	59	70	71	79	75	71	69
Diagnosis	SiNET	SiNET	SiNET	PNET	SiNET	SiNET	SiNET	SiNET
Pelvic bone metastasis	Yes	Yes	No	No	Yes	Yes	No	Yes
Baseline GFR (mL/min)	70	84	79	101	66	50	78	65
Previous Therapy	No	CT°	No	CT, TT	No	EBRT	EBRT	CT°, EBRT
Cumulative 177LulLu-DOTATATE activity (GBq)	28.6	14.7	22.3	21.4	22.2	29.5	28.8	12.6
Osteo-Medullary Invasion Score OMIS (%)	41.5	90	0	0	36	70	0	59.5
Spleen Length (23)	85	110	103	124	72	53	56	59
Baseline platelet count (10^9^/L)	173	144	122	108	300	228	233	167
Initial Decline of Platelet Count IDPC (%)	32	61	34	31	68	38	26	54
Nadir platelet count during PRRT (10^9^/L)	57	53	81	62	37	95	99	43
Platelet count 2 months post PRRT (10^9^/L)	65	70	92	73	49	95	99	43
Platelet count 12 months post PRRT (10^9^/L)	80	43	99	81	113	160	120	5
Time between end of PRRT and death (months)	42	12	20	35	38	37	18	11

SiNET, small intestine neuroendocrine tumors; PNET, pancreatic neuroendocrine tumors; GFR, glomerular function rate; CT, chemotherapy; C°, chemotherapy with alkylating agent; TT, targeted therapy; EBRT, external-beam radiation therapy.

Regarding the early platelet toxicity of the global population during PRRT, 17 patients had grade 1 thrombocytopenia (36.2%), 3 patients grade 2 (6.4%), and 2 patients grade 3 (4.2%). The median IDPC was 26% (min -1; max 68) and 19 patients (40.4%) presented with an IDPC superior to 30%.

Two months after the end of PRRT, 14 patients had grade 1 (29.8%) thrombocytopenia, 3 patients grade 2 (6.4%), and 2 patients grade 3 (4.2%). The median platelet count at this time was 162 x 10^9^/L (range 43-394).

### Univariate analysis

#### Baseline potential predictive factors for PT

The occurrence of PT was unrelated to the following patient-related parameters: age, gender, primary tumor, tumor grade, renal function, presence of bone metastases, previous systemic therapies (chemotherapy, targeted therapy), and previous cancer. Previous EBRT was slightly associated with PT occurrence with a statistical tendency [OR=5.3, 95% confidence interval (0.9–30.7), p=0.070]. Among the seven patients who had previous EBRT, three developed PT. The presence of pelvic bone metastases was significantly associated with PT [OR=7.6 (1.5–39.6), p=0.016]. Patients with pelvic bone metastases developed PT in 41.7% of cases, compared with 8.6% among those without pelvic bone involvement. An OMIS ≥ 30% was significantly associated with PT [Fisher’s exact test p<0.001, modified Wald OR=124.1 (5.6–2,740.7)]. All of the patients with an OMIS ≥ 30% (5/5) developed a PT vs. 7.1% of patients with an OMIS<30%. A spleen length larger than 100 mm was associated with PT [OR=7 (1.1–44.7), p= 0.040]. Half of the patients with a large spleen developed PT (3/6). The baseline median platelet count was significantly lower in the group of patients who developed PT, 170 x 10^9^/L compared with 230 x 10^9^/L in the group without PT (Wilcoxon test, p=0.030). This factor was significantly associated with PT occurrence [OR=0.98 (0.96–0.99), p=0.020] in its quantitative form, with an AUC=0.75 [95% confidence interval (0.53–0.98)].

#### Potential predictive factors for PT from PRRT follow-up

**Figure 3:**
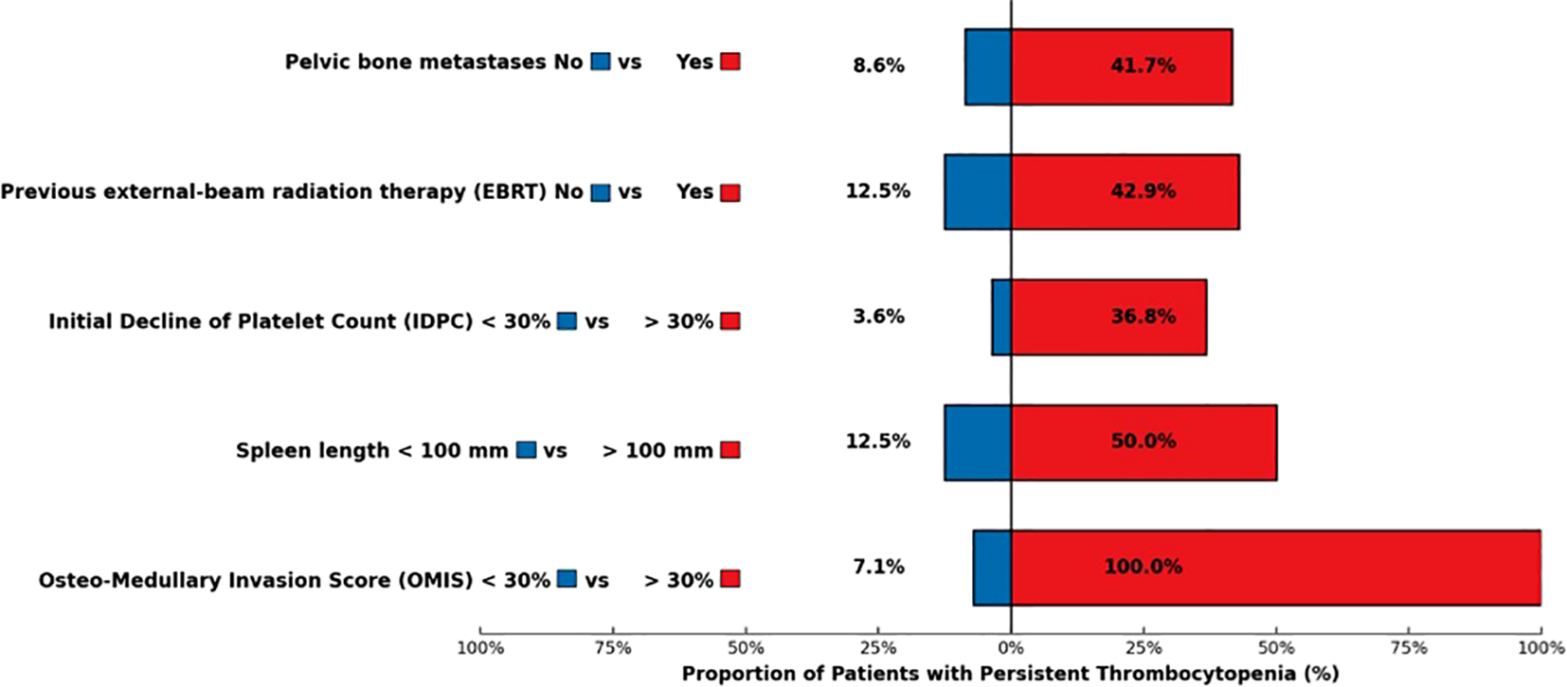
Predictive factors of Persistent Thrombocytopenia (PT) post-PRRT.

An IDPC ≥30% was significantly correlated with PT [OR=15.8 (1.7–142.5), p=0.014]. Among the eight patients with PT, seven of them had an IDPC ≥ 30%. Cumulative activity and number of PRRT cycles were not associated with PT. In the group of patients with PT, only 37.5% received the planned four cycles of PRRT vs. 80.6% in the global population. The most relevant results of this univariate analysis are shown in **Table 3** and **Figure 3**.

**Table 3:** Persistent thrombopenia occurrence - univariate analysis.

Characteristic	Event (%)	OR [95% CI]	p*
Previous external-beam radiation therapy (EBRT)			
No	5/40 (12.5)		
Yes	3/7 (42.9)	5.3 [0.9-30.7]	0.066
Pelvic bone metastases			
No	3/35 (8.6)		
Yes	5/12 (41.7)	7.6 [1.5-39.6]	0.016
Osteo-Medullary Invasion Score (OMIS)			
< 30%	3/42 (7.1)		
30%	5/5 (100)	124.1 [5.6-2470.7]	<0.001
Spleen length			
< 100 mm	5/40 (12.5)		
100 mm	3/6 (50)	7 [1.1-44.7]	0.04
Median platelet count before PRRT X 10^9^/L Initial Decline of Platelet Count (IDPC)		0.98 [0.96-0.99]	0.022
< 30%	1/28 (3.6)		
30%	7/19 (36.8)	15.8 [1.7-142.5]	0.014

OR, Odds Ratio; CI, bilateral Wald Confidence Interval, *p-value for Wald test for significance in univariate logistic model, or exact Fisher test in case of non-estimable OR.

## Discussion

PRRT with [177Lu]Lu-DOTATATE is a well-established treatment for patients with metastatic NETs, particularly those progressing after first-line therapies. While hematological toxicity is a known adverse effect, it is often considered mild and reversible, with a nadir typically occurring 4 to 6 weeks post-treatment. However, the persistence of cytopenia in some cases can delay or limit subsequent therapies, affecting overall patient management (3, 17).

Retrospective studies report that approximately 10% of patients experience grade 3/4 subacute hematological toxicity following [177Lu]Lu-DOTATATE, including anemia, neutropenia, and thrombocytopenia (6). Among these, grade 3/4 thrombocytopenia alone occurs in approximately 5% of cases (18). In some patients, cytopenia may persist for more than 6 months. Ezzidin et al. described a median time to complete bone marrow recovery of 11 months (range: 3–18) following PRRT cessation (19). Del Prete et al. reported a chronic grade 1/2 hematological toxicity affecting the platelet lineage in 47.6% of patients (21 patients assessable for chronic toxicity) (20). Despite a focus on severe cytopenia in the literature, little is known about prolonged grade 1/2 thrombocytopenia, which may still impair treatment strategies and worsen prognosis (6, 18–20).

In the NETTER-1 trial, myelosuppression occurred more frequently in patients receiving [177Lu]Lu-DOTATATE compared to those receiving high-dose octreotide (3). While the NETTER-1 trial reported a 25% incidence of thrombocytopenia (all grades), the U.S. Summary of Product Characteristics for Lutathera^®^ describes a 53% rate (21). This difference may stem from broader pharmacovigilance data or methodological variations in adverse event reporting. Such variations underline the complexity of accurately characterizing hematological toxicity and the value of continued post-approval safety monitoring. Grade 3/4 thrombocytopenia was observed in 1% vs. 0%, and platelet nadir occurred at a median of 5.1 months. Of the 59 patients who developed thrombocytopenia, 68% recovered to baseline levels, with a median recovery time of 2 months, and 32% presented with PT, mostly grade 1/2. These findings underscore the variability in hematological toxicity and highlight the need for better predictors. Importantly, even grade 1 thrombocytopenia can be clinically meaningful when prolonged, as it may preclude the initiation of chemotherapy or targeted therapy in patients with progressive disease post-PRRT (3, 21).

In our retrospective cohort, we aimed to identify predictors of PT. Thrombocytopenia < 100 x 109/L is considered a grade 1 CTCAE but can be relevant in the case of persistence for months after the end of PRRT. We chose this cut-off since it seemed to be widely considered for the prescription of anti-tumor treatment using chemotherapy or targeted therapy in case of progressive disease post-PRRT. We observed a 17% incidence of PT and 36.2% of grade 1/2 platelet toxicity at 2 months post-PRRT. Grade 3 thrombocytopenia occurred in 4.2% of patients, without any grade 4 cases. We focused on three main predictive factors: bone marrow infiltration (quantified as OMIS), spleen length, and early platelet count dynamics (IDPC) (present study,14).

Unlike most studies that report bone metastases qualitatively, we used a quantitative imaging score, the OMIS. We found that an OMIS ≥ 30% was significantly associated with PT. This highlights the added value of quantifying bone involvement, as high OMIS scores may reflect a compromised hematopoietic reserve. Although OMIS was calculated manually, ongoing work aims to automate this process using AI tools. In clinical practice, OMIS could be used to stratify patients by risk and adapt PRRT planning accordingly (10, 12, 14).

The spleen shows very high uptake of somatostatin analogs, and the red pulpa of the spleen is a site of somatostatin analog receptor expression (22, 23). Spleen uptake of somatostatin analogs is high during PRRT, and the spleen receives one of the highest organ doses (24). Additionally, the spleen is a major site of extramedullary hematopoiesis in the context of cancer (25). Recently, Hebert et al. showed a correlation between the decrease of hematologic parameters such as lymphocytes or platelet concentrations and the absorbed doses by the spleen or bone marrow (26). Morland et al. found that the mean spleen SUV allowed them to predict subacute anemic complications after 177Lu-DOTATATE therapy (27). Nevertheless, Kulkarni et al. did not observe any correlation between the incidence or grade of hematological toxicity and the dose to the spleen (28). While previous studies have described a protective effect of splenectomy against hematological toxicity (6, 13), we could not verify this due to limited cases. However, we observed a significant correlation between spleen length >100 mm and PT, suggesting spleen size may serve as a simple, non-invasive biomarker. This is, to our knowledge, the first such description (22–28).

The identification of predictive factors such as OMIS and spleen length raises the important question of how these variables could be translated into clinical decision-making. In our view, these markers could be used to stratify patients into risk categories prior to PRRT initiation. For instance, a high OMIS score or a spleen length >100 mm may prompt clinicians to implement closer hematological monitoring, consider interval prolongation between cycles, or adapt the number of PRRT administrations. In borderline cases, these factors could support shared decision-making with patients and oncology teams regarding the benefit-risk ratio of continuing full-course therapy. While our results are preliminary, they suggest a potential role for such parameters in personalizing PRRT delivery. Prospective validation in larger cohorts is warranted before these factors can be formally integrated into treatment algorithms.

In addition to these imaging biomarkers, we found that an early decrease in platelet count between baseline and pre-cycle 2 (IDPC ≥ 30%) was significantly correlated with subsequent PT. This early hematological marker could serve as a practical and accessible predictor of long-term toxicity, offering clinicians a window of opportunity to adjust treatment before more severe or prolonged cytopenia occurs. This finding aligns with previous studies showing that early hematological changes can predict later toxicity. Thus, early biological follow-up could serve as a valuable and timely tool for physicians to anticipate complications and adjust therapy (6, 20).

Interestingly, other expected variables, such as age, renal function, prior systemic therapies, and cumulative [177Lu] Lu-DOTATATE activity, were not associated with PT in our population. In 125 patients with pancreatic NETs who were heavily pretreated with chemotherapy, Frosse-Baron et al. showed that [177Lu] Lu-DOTATATE bone marrow toxicity was related to neither type and duration of previous chemotherapy nor to PRRT parameters (administered activity, number of cycles and bone marrow dose) (29). This observation supports findings from other retrospective cohorts (6, 9, 18) and suggests that patient-specific factors beyond cumulative dose may drive hematological vulnerability. A further argument for this inverse correlation is the safety of salvage therapy with extra cycles of PRRT without severe hematological side effects (30, 31). In fact, the apparent inverse correlation between cumulative activity and PT (with only 37.5% of PT patients completing all four planned cycles vs. 80.6% in the global cohort) likely reflects early discontinuation due to toxicity, rather than a protective effect of higher dosing. It is thus conceivable that patients tolerating full treatment courses may inherently have lower risk profiles (29–31).

Among the eight patients (five men and three women) with PT, we did not observe sex-related differences, however, Minczeles et al. recently identified more severe platelet and hemoglobin toxicities in female compared to male patients with NETs (32).

Regarding long-term hematological events, one patient (2.1%) developed a complex hematological malignancy (myelodysplastic syndrome with associated multiple myeloma) 40 months after PRRT, which is consistent with previously reported rates (3, 9, 33). Two other patients experienced prolonged bone marrow hypoplasia without progression to malignancy. All three had PT persisting beyond 1 year. While no deaths were directly attributable to thrombocytopenia, the long-lasting cytopenia likely reduced therapeutic options and may have impacted survival. These cases highlight the need for long-term monitoring in patients with persistent cytopenia (3, 9, 33).

## Limitations

Our study has limitations, most notably the small sample size and the absence of dosimetry. In addition, the use of post-PRRT [177Lu]Lu-DOTATATE scintigraphy, rather than more sensitive imaging modalities, such as 68Ga-DOTATOC PET, may have underestimated bone marrow involvement. The retrospective design also limited our ability to assess causal relationships and precluded systematic longitudinal hematological follow-up. Due to the limited number of patients with PT (n = 8), multivariate analysis could not be reliably performed. This small sample size restricted our statistical approach to univariate analyses only, despite the exploratory nature of the study and its continuity with previous work.

## Conclusion

In conclusion, this study underscores the importance of identifying early predictors of hematological toxicity in patients undergoing PRRT. Quantitative assessment of bone marrow infiltration and early decreases in platelet count between treatment cycles may help clinicians anticipate and mitigate PT. Spleen size may also serve as a simple and novel biomarker of hematological risk. Further validation in larger cohorts with a prospective design and integrated dosimetry is necessary to confirm the predictive value of the identified factors and develop predictive models that can guide individualized treatment strategies.

## Data Availability

The datasets presented in this study can be found in online repositories. The names of the repository/repositories and accession number(s) can be found below: ferrarar@ipc.unicancer.fr.
